# Neurodevelopmental disorders in south Asia: closing the evidence gap

**DOI:** 10.1016/j.lansea.2026.100716

**Published:** 2026-01-16

**Authors:** The Lancet Regional Health – Southeast Asia

With an estimated 659 million children living in the region, south Asia is central to any discussion on child and adolescent health. Over the last two decades, improvements in child survival have shifted the burden of disease from acute to chronic conditions associated with long-term disability. Among these, conditions impacting neurodevelopment, or neurodevelopmental disorders (NDDs)—including autism spectrum disorder (ASD), attention-deficit hyperactivity disorder (ADHD), developmental learning disorders, and developmental speech and language disorders—present early and affect multiple developmental domains, shaping health, education, and social participation across the life course. More recently, NDDs are increasingly understood as neurodevelopmental differences, usually present from birth relating to a diverse way of being, requiring understanding and support to reduce related adversity and disability.

However, NDDs remain poorly characterised within south Asia. According to Global Burden Of Disease data (2021), the global number of cases for three relatively common NDDs—ASD, ADHD, and idiopathic developmental intellectual disability—was estimated at approximately 235 million. These numbers suggest that NDDs account for a substantial proportion of years lived with disability in children and adolescents globally. In south Asia, these estimates rely largely on modelling or pooled prevalence estimates due to the scarcity of region-specific primary data. Reliable epidemiological data on the prevalence and distribution of NDDs are notably lacking across the region. Existing studies are frequently hospital-based, geographically limited, and methodologically heterogeneous, limiting their generalisability and our understanding of the real picture in the region. Children in rural areas, those not enrolled in school, and those from socially marginalised communities are particularly likely to be under-represented. Larger datasets from the region such as the National Mental Health Survey of India, conducted between 2015 and 2016, did not incorporate NDDs into the survey framework. Establishing population-based surveillance and embedding neurodevelopmental outcomes within existing maternal and child health cohorts should therefore be a core research priority.

Evidence gaps in diagnosis further constrain both research and clinical care. In spite of some standout tools like the Indian Scale for Assessment of Autism (ISAA), diagnostic tools commonly used for NDDs were largely developed in non-regional settings and have undergone limited validation across south Asian languages, cultures, and educational contexts. Translation is often assumed to be sufficient, despite differences in social expectations of child behaviour and communication. Clinical management is similarly shaped by non-local evidence. Comparative studies embedded within routine services are urgently needed. Furthermore, comorbidity between NDDs and other neuropsychiatric conditions affects the majority of individuals with NDDs. However, much research continues to examine single diagnoses in isolation, producing evidence that poorly reflects clinical reality. In a study published in *The Lancet Child & Adolescent Health*, the authors found that more than half of children enrolled who were living with autism were also found to have ADHD.

Integrated approaches addressing multimorbidity are particularly relevant in south Asia, where care pathways are fragmented and access to multidisciplinary specialist services is limited, indicating a substantial care gap. This is a key driver of the limited research focus in the region. A recent WHO report highlighted a significant shortage in trained mental health-care professionals and inadequate access to specialist care in the region. Initiatives such as the WHO caregiver skills training programme, piloted in 30 countries including India and Indonesia, offer an opportunity to generate robust evidence on caregiver-led models of care, which often represent the most sustainable and accessible interventions available to children across the region. Similarly, community-based programmes such as ADD International Bangladesh and Atmiyata in India demonstrate the potential of peer-led support models, which should be taken as examples for research into community-based programmes in policy and should inform future policy-oriented research.

Many of these gaps also reflect broader structural challenges, including fragmented research efforts, limited funding for locally led studies, and the under-representation of south Asian populations in global neurodevelopmental research collaborations. Strengthening regional research networks are essential to ensure that evidence generation translates into meaningful improvements in care. *The Lancet Regional Health – Southeast Asia* recognises the urgent need for high-quality, regionally grounded research to inform practice and policy that may impact the lives of millions of young people in the region. We therefore invite robust primary research from multi-disciplinary teams that are actively involved in the day-to-day care and support of impacted children, including paediatric neurology, developmental paediatrics, and allied health services, to contribute to shaping the clinical decision making and care pathways for young children. It is time for action that aligns research, care pathways, and policy to secure better futures for children and families across the region.

